# RNA Interference Past and Future Applications in Plants

**DOI:** 10.3390/ijms24119755

**Published:** 2023-06-05

**Authors:** Sarah Koeppe, Lawrence Kawchuk, Melanie Kalischuk

**Affiliations:** 1 Department of Plant Agriculture, University of Guelph, 50 Stone Road E., Guelph, ON N1G 2W1, Canada; 2 Research Centre, Agriculture and Agri-Food Canada, 5403 1 Ave S., Lethbridge, AB T1J 4B1, Canada

**Keywords:** RNAi, immunity, HIGS, VIGS, SIGS

## Abstract

Antisense RNA was observed to elicit plant disease resistance and post-translational gene silencing (PTGS). The universal mechanism of RNA interference (RNAi) was shown to be induced by double-stranded RNA (dsRNA), an intermediate produced during virus replication. Plant viruses with a single-stranded positive-sense RNA genome have been instrumental in the discovery and characterization of systemic RNA silencing and suppression. An increasing number of applications for RNA silencing have emerged involving the exogenous application of dsRNA through spray-induced gene silencing (SIGS) that provides specificity and environmentally friendly options for crop protection and improvement.

## 1. Introduction

RNA silencing is a revolutionary innate immunity mechanism in eukaryotes that has greatly expanded our knowledge of gene expression and regulation in plants. RNA interference (RNAi) is an important regulatory mechanism that has become an invaluable tool for plant research, especially in terms of understanding the effects of gene regulation in response to abiotic and biotic stress. RNAi has enabled researchers to gain insight into gene function, pest resistance, and physiological processes in plants. Although RNA is known to play critical roles in biology, the extensive capabilities and complexity of this nucleic acid remained elusive and not fully understood. The intrinsic nature of the relatively labile, often single-stranded RNA molecule, and limited availability of RNA-dependent enzymes had slowed characterization and progress. Traditional research focused on messenger transcript (mRNA), transfer RNA (tRNA), and ribosomal RNA (rRNA), but the universality of the molecule to life remained underestimated [1]. The occurrence of viruses with RNA genomes (gRNA), the dominant genome type of viruses in plants, provided an extraordinary platform for the study of RNA function and gene expression. In a relatively brief period of time, knowledge of the dynamic RNA molecule has dramatically increased, and our understanding of RNA capabilities and applications rapidly expanded. This review summarizes key developments in the discovery and characterization of RNAi and examines applications in plants to advance agricultural biotechnology, crop engineering, pest control, and virus resistance.

Plant viruses can cause major diseases in crops worldwide. Management has relied on a combination of strategies involving virus eradication and transmission prevention. Early studies reported the occurrence of cross-protection in plants where infection with a mild strain of a virus protected against infection by more pathogenic strains [2]. A major advance based on this phenomenon, and the suggestion that introduction of the viral coat protein may provide protection, was the resistance observed with the introduction of the Tobacco mosaic virus (TMV) coat protein mRNA into transgenic tobacco [3]. Several subsequent studies reported a similar resistance for other groups of plant single-stranded RNA (ssRNA) viruses including the family *Solemoviridae*, one of the most devastating group of viruses worldwide for many important crops [4,5]. Molecular characterization and control of *Solemoviridae* has been especially challenging as they are phloem-limited and transmitted in a persistent circulative manner by specific aphids.

## 2. RNA Interference

**Figure 1:**
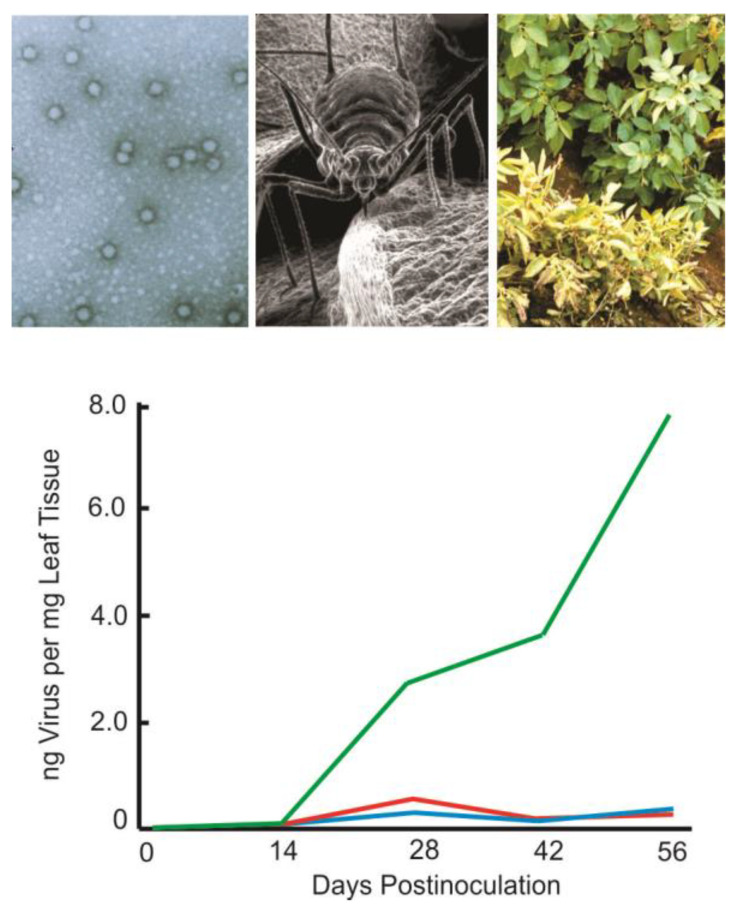
RNA interference (RNAi) against virus infection. Sense and antisense RNA protection against the single-stranded positive-sense RNA Potato leafroll virus (PLRV). Although phloem limited, members of the genus *Polerovirus* are transmitted in a persistent, non-propagative manner and cause considerable disease-related losses worldwide. The icosahedral virus is approximately 25 nm in size (top left transmission electron photomicrograph) is transmitted in an aphid-specific manner by the green peach aphid, *Myzus persicae,* approximately 2.5 mm in length (top middle scanning electron photomicrograph). Disease symptoms include stunting and chlorosis of infected plants (top right) that reduce yield and quality. Virus titres in plants expressing coat protein messenger RNA (mRNA, red line) or antisense RNA (aRNA, blue line) reduced virus levels significantly as compared to untransformed controls (green line), determined by double antibody sandwich (DAS) enzyme-linked immunosorbent assay (ELISA).

Remarkably, transformation of plants with the genus *Polerovirus* coat protein antisense RNA of the Potato leafroll virus produced similarly high levels of reduced virus titre and disease resistance as the corresponding sense mRNA [4,6]. The response was rapid and all transformed plants exhibited sequence-specific sustained high levels of immunity regardless of the virus inoculum concentration (Figure 1). Vector transmission of the virus by the green peach aphid *Myzus persicae* was reduced and disease symptoms in foliage and tubers were eliminated. This showed that RNA was capable of conferring resistance as a trigger molecule and was subsequently observed in other plant virus groups [7]. Replicative intermediates of RNA viruses include double-stranded RNA (dsRNA) and dsRNA secondary structures that are produced to regulate gene expression and are relatively stable compared to ssRNA due to the widespread occurrence of resilient ssRNA ribonucleases [8]. The disease resistance achieved with antisense RNA demonstrated an inherent ability of RNA to protect against pathogens.

Experiments to transiently or stably increase endogenous gene expression often unexpectedly produced a decrease in mRNA. For example, attempts to overexpress chalcone synthase (CHS) in pigmented petunia petals blocked anthocyanin biosynthesis [9]. Developmental timing and expression of the CHS mRNA by the endogenous gene was not altered but the level of transcript was reduced by 50 fold. This posttranslational gene silencing (PTGS) highlighted a regulatory mechanism of gene expression involving RNA interference. Polygalacturonase involved in plant cell wall degradation and ripening was inhibited in transgenic tomato expressing antisense RNA [10]. Similarities between viral defense and gene silencing mechanisms suggested a common innate immunity in plants, including the systemic signalling in gene silencing contributing to the sequence-specific RNA interference [11,12].

## 3. Characterization of RNA Interference

RNA interference (RNAi) involves a sequence-specific suppression of gene expression by transcriptional or translational repression. The results of the RNAi characterization demonstrated that feeding or injecting gene-specific dsRNA into *Caenorhabditis elegans* resulted in the disappearance of the targeted message [13]. Silencing effects were observed with only a few molecules of *unc-22* dsRNA per cell supporting a role as a trigger molecule. The RNAi mechanism is a naturally occurring process in most eukaryotes, conferring an ability of dsRNA to induce a sequence-specific systemic silencing process [6,9,10,13]. 

**Figure 2:**
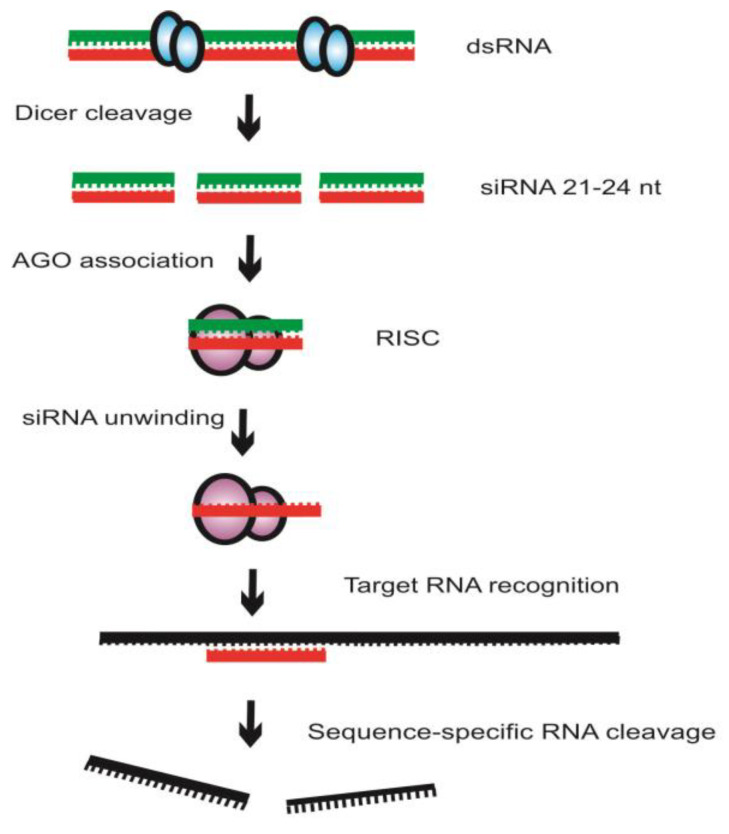
Mechanism of RNA interference (RNAi). RNAi is initiated by the enzyme Dicer that cleaves double-stranded RNA (dsRNA) into short fragments of approximately 21- to 24-nucleotide short interfering RNA (siRNA). The siRNA is unwound into single-stranded RNA and the sense RNA (green) is further cleaved and degraded by the enzyme Argonaute (AGO). The antisense RNA (red) is recruited into the RNA-induced silencing complex (RISC) that binds to the target sense RNA through the specificity of the complementary antisense RNA.

Exogenous dsRNA initiates RNAi by activating the ribonuclease Dicer enzymes that bind and cleave dsRNA into 21–24-base-pair small interfering RNA (siRNA) fragments with 3′ overhangs of 2–3 nucleotides (Figure 2). Dicer proteins have an RNA helicase domain, RNase III motifs, and nucleic-acid-binding PAZ domain [7]. The siRNA is converted to ssRNA when the sense complimentary RNA strand is degraded by Argonaute (AGO) enzymes and the antisense guide strand is incorporated into the RNA-induced silencing complex (RISC). Members of AGO possess a PAZ domain and a PIWI domain, resembling RNaseH, that are required for cleavage activity [7]. The RISC complex further uses this strand to bind and degrade additional copies of sense complimentary RNA. Systemic silencing occurs and the inherit specificity suggests that nucleic acid is the signal molecule in plants [7,12]. Amplification of even weak silencing signals indicates that RNA-dependent RNA-polymerase (RDRP) recognition and replication elicits effective silencing.

The occurrence of double-stranded RNA during viral RNA replication and hairpin RNA secondary structures regulating gene expression, indicated that ssRNA viruses have an inherent protective mechanism from RNAi [14,15]. Silencing suppressors were subsequently identified within RNA virus genomes that targeted different components of RISC, such as the DICER-LIKE (DCL) proteins, and inhibit innate RNA silencing [16]. Similar to the systemic nature of RNAi, silencing suppressors were also capable of systemic silencing suppression [17]. The application of RNA silencing suppressors, such as the Tomato bushy stunt tombusvirus p19 protein, are often required in preventing PTGS in plant studies expressing homologous or heterologous genes [14]. 

## 4. Applications of RNA Interference

**Figure 3:**
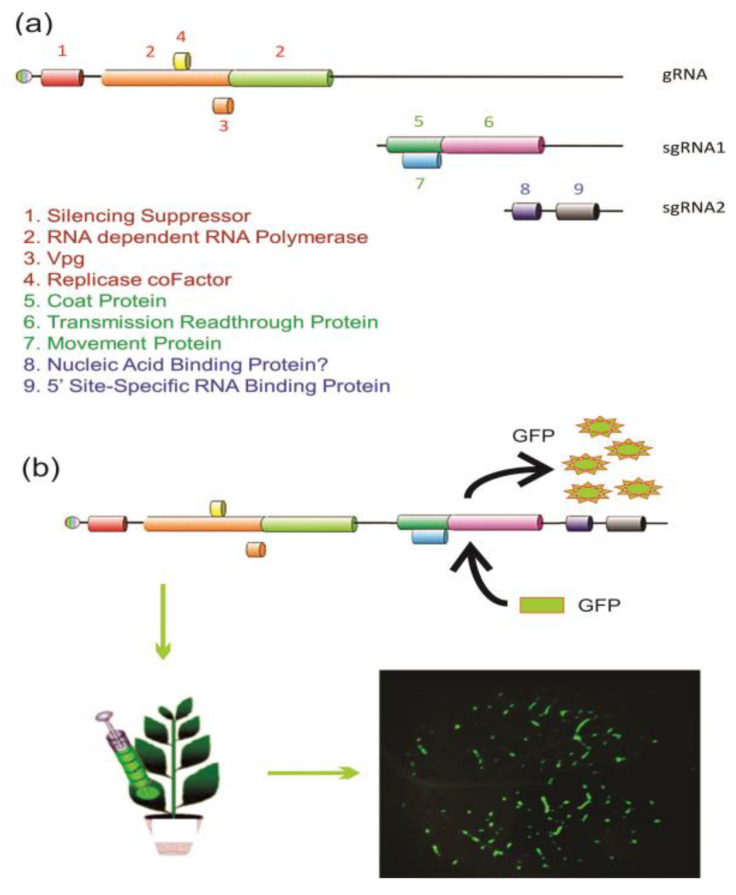
RNA virus replication and applications. (**a**) Genomic and subgenomic RNA for the replication and translational strategies of the Potato leafroll virus, including a silencing suppressor produced immediately following virus disassembly. Replication involves the production of antisense RNA and subsequent sense subgenomic RNAs (sgRNAs) and expression of proteins involves several translational strategies including leaky start and stop codons, proteolytic site-specific cleavage of genomic RNA (gRNA), and an internal ribosomal entry site (IRES) sequence. (**b**) A full-length infectious clone (FLIC) of the Potato leafroll virus RNA amplifies expression of heterologous sequences for virus-induced gene silencing (VIGS) or production of commercially valuable proteins as shown (magnification 0.25×) with green fluorescent protein (GFP).

Stably or transiently expressed genes and nucleic acids in genetically engineered plants is often utilized in the study of gene function or the heterologous production of commercially valuable products. The use of full-length infectious clones (FLICs) of RNA viruses has facilitated the amplification of targeted genes, providing a convenient vector platform that can circumvent RNAi for site-directed mutations and increase or reduce gene expression to characterize PTGS and produce valuable heterologous commercial products. Application of virus-induced gene silencing (VIGS) has successfully utilized several RNA virus vectors including Tobacco rattle virus (TRV), Potato virus Y (PVY), TMV, and PLRV [18,19,20,21]. Different virus vectors confer specific advantages such as titre and tissue specificity. For example, field-grown plants are subjected to strict containment by regulatory agencies to limit unexpected transmission in the environment by vectors. Phloem-specific expression by the PLRV FLIC is not transmitted mechanically or by vector when the capsid readthrough protein is replaced by the heterologous nucleic acid, eliminating accidental movement of genetic materials (Figure 3). 

Innate immunity is more complex than originally envisioned and the RNAi regulatory mechanism is independent of other recognition and signalling pathways. Identification of genes for gene receptors and avirulence proteins has advanced our understanding of cellular resistance to a wide range of pathogens, including *Pseudomonas syringae*, *Cladosporium fulvum*, and *Verticillium* species [22,23,24,25]. Mechanisms for signal amplification and recognition by receptors of sessile plants has improved our understanding of an important component of innate immunity [26,27]. Cross-protection and intracellular communication has expanded with the discovery of RNAi and its role in innate immunity and gene regulation through extracellular plant and fungal RNA [28,29]. Together the different sources of innate immunity provide complementary strategies in controlling historically devastating crop losses and emerging new threats to food production.

Exogenously introduced dsRNA to target plant pests began with the introduction of dsRNAs through microinjections [30,31]. Microinjections are a favoured laboratory technique because incredibly precise amounts of dsRNA can be introduced into the target organism, allowing for precise delivery [32]. Although an adequate delivery method for lab- and smaller-scale applications, microinjection unfortunately is not suitable for field-level control of plant pests and pathogens [30,31]. Another delivery method involves the soaking of an organism in a suspension that contains the target dsRNA or directly spraying it with a solution containing the dsRNA [32]. This method may not be as exact as microinjection; however, it is often used because of its ease of use and overall convenience. Many other methods of RNA delivery have been examined and application choice is often influenced by several factors including efficacy and economics (Table 1). 

Investigators have created transgenic plants that express desired dsRNAs to cause RNAi-induced gene silencing in the target organism when it ingests plant material, referred to as Host-Induced Gene Silencing (HIGS) [31,33]. One example of HIGS was transgenic *Zea mays* (corn) called SmartStax Pro that was created to target corn rootworm (*Diabrotica vwirgifera virgifera*) that was approved for commercial use by the U.S. Environmental Protection Agency, the U.S. Food and Drug Administration, and the U.S. Department of Agriculture [31,33,34]. Commercial acceptancet of transgenic plants has been challenging due to general public concerns related to genetic engineering, especially the stable insertion of nucleic acid from other organisms [33,35]. A similar pest control efficacy was achieved with exogenously applied dsRNA in plants, representing a friendlier environmental and regulatory strategy for protection and production improvements [30].

Microorganisms transformed to contain target dsRNA have also been evaluated as a method for exogenous application. One notable example showed that bacteria transformed to contain the target dsRNA could be fed to insects to induce RNAi [36]. These genetically modified bacteria in some cases were even able to colonize the gut of the host and continue to deliver dsRNA directly to it through the gut. Another example of the ingestion of a transformed microorganism is the study of transformed *Saccharomyces cerevisiae* (yeast) containing dsRNA targeting spotted wing fruit fly *Drosophila suzukii* [37]. This type of yeast naturally occurs on the surface of rotting fruit that *D. suzukii* consumes, and therefore was seen as a viable vector to induce oral ingestion of the dsRNA. They had success and found that locomotor activity, survivorship, and reproductive fitness were all negatively impacted by the complimentary dsRNA [37]. Acceptance of products derived from transgenic platforms are subjected to elevated regulatory and consumer acceptance concerns.

**Table 1:** Examples of spray-induced gene silencing of different targets and organisms.

Viral/Viroid Target	Host	dsRNA Target	Application Method	Reference
Sugarcane Mosaic Virus (SCMV)	Corn	Coat protein	Escherichia coli HT115 co-inoculation spray with dsRNA-producing bacteria	[38]
Pea Seed-borne Mosaic Virus (PSbMV)	Pea	Coat protein	dsRNA spray	[39]
Pepper Mild Mottle Virus (PMMoV)	Tobacco	PMMoV replicase	Spray containing dsRNA coated in Layered Double Hydroxide (LDH) bioclay	[40]
	Tobacco, pepper	RP gene (multiple lengths)	Tobacco leaves treated with carborundum, dsRNA mixed with inoculum rubbed on leaves	[41]
	Tobacco	RP gene	Mechanical inoculation of dsRNA with co-inoculation using an atomizer	[42]
Cucumber Mosaic Virus (CMV)	Cowpea	CMV 2b	Spray containing dsRNA coated in Layered Double Hydroxide (LDH) clay nanosheets	[40]
Bean Common Mosaic Virus (BCMV)	Tobacco	Nuclear inclusion b protein (Nib) and coat protein	dsRNA mechanical inoculation with carborundum sprayed with an atomizer	[43]
Tomato Yellow Leaf Curl Virus (TYLCV)	Tobacco, tomato	Coat protein	LDH clay nanosheets sprayed using an atomizer	[44]
Tobacco Mosaic Virus (TMV)	Tobacco	Coat protein and TMV p126	dsRNA (virus co-inoculated rubbed on carborundum-dusted tobacco leaves	[45]
	Tobacco	TMV replicase, movement protein	Tobacco leaves were dusted with celite, and dsRNA solution was rubbed on	[46]
	Tobacco	Coat protein	dsRNA and purified TMV solution inoculation	[47]
Tobacco Etch Virus (TEV)	Tobacco	RP gene (multiple lengths)	Tobacco leaves treated with carborundum, dsRNA mixed with inoculum rubbed on leaves	[41]
Alfalfa Mosaic Virus (AMV)	Tobacco	RP gene (multiple lengths)	Tobacco leaves treated with carborundum, dsRNA mixed with inoculum rubbed on leaves	[41]
Papaya Ringspot Virus (PRSV)	Papaya	Coat protein	dsRNA in PRSV-infected papaya sap that was mechanically inoculated	[48]
Isolates Tirupati and Delhi	Papaya	Coat protein and HC-Pro	dsRNA in PSRV-infected papaya sap rubbed on to leaves dusted with carborundum	[49]
Cymbidium Mosaic Virus (CymMV)	Brassolaeliocattleya hybrida	Coat protein	Celite-treated orchid leaves were inoculated with dsRNA in the form of crude bacterial lysate, and gently rubbed onto said leaves	[50]
Zucchini Yellow Mosaic Virus (ZYMV)	Watermelon, cucumber, squash	Helper component proteinase (Hc-Pro) and coat protein	dsRNA in ZYMV-infected summer squash sap gently rubbed on to carborundum-dusted leaves	[51]
Potato Spindle Tuber Viroid (PSTVd)	Tomato	Viroid-specific gene	dsRNA rubbed on to leaves that were dusted with carborundum	[52]
Chrysanthemum Chlorotic Mottle Viroid (CChMVd)	Tomato and chrysanthemum	Viroid-specific gene	dsRNA rubbed on to leaves that were dusted with carborundum	[52]
Citrus Exocortis Viroid (CEVd)	Tomato and Gynura	Viroid-specific gene	dsRNA rubbed on to leaves that were dusted with carborundum	[52]
**Fungal Target**	**Host**	**dsRNA Target**	**Application Method**	**Reference**
*Fusarium graminearum*	Barley	Cytochrome P450 lanosterol, C-14α-demethylases CYP51A, CYP51B, CYP51C	dsRNA sprayed on detached barley leaves	[53]
	Arabidopsis and Barley	FgCYP51A, FgCYP51B, and FgCYP51C in various combinations	Detached leaves sprayed with naked dsRNA	[54]
	Barley	DCL1 and DCL2, AGO1 and AGO2, AGO-interacting protein, FgQIP, RecQ helicase, several Fg RNA-dependent RNA polymerases	dsRNA sprayed on detached leaves	[55]
	Barley	AGO1 and AGO2, DCL1 and DCL2 in combinations	dsRNA sprayed on detached leaves	[56]
	Barley	CYP51A, CYP51B, CYP51C	Detached barley leaves drop-inoculated with dsRNA	[57]
*Fusarium asiaticum*	Wheat	Myosin5	dsRNA sprayed on plant surface	[58]
	Wheat	Faβ2Tub-3	Naked, sprayed dsRNA	[59]
*Fusarium oxysporum*	Tomato	CYP51, chitin synthase 1, elongation factor 2	LDH nanosheet-coated dsRNA spray on leaf	[60]
*Fusarium oxysporum* f. sp. *cubense*	Banana	Nuclear condensin, coatomers alpha and zeta, DNA-directed RNA polymerase, ARP 2/3, cap methyltransferase, proteasome Pre4, ribosomal RNA, DNA polymerase alpha and delta subunits, adenyl cyclase, protein kinase C, FRQ RNA helicase	dsRNA aliquoted into spore suspension	[61]
*Magnaporthe oryzae*	Barley	Faβ2Tub-3	Naked, sprayed dsRNA	[59]
*Colletotrichum truncatum*	Soybean	Faβ2Tub-3	Naked, sprayed dsRNA	[59]
*Botrytis cinerea*	Arabidopsis, tomato, grape, rose, lettuce, onion, strawberry	Dicer-like DCL1 and DCL2	dsRNA co-inoculated on fruits, vegetables, and rose petals	[62]
	Cucumber	Faβ2Tub-3	dsRNA sprayed on plant surface	[59]
	Grapevine	BcCYP51, BcCHS1, BcEF2	dsRNA sprayed on plant surface	[63]
	Tomato, lettuce, rose, and grape	DCTN1, SAC1, DCL1, and DCL2	Drop inoculation of dsRNA for lettuce, rose, tomato fruit, and grape, dsRNA spray for tomato leaves	[64]
	Rapeseed	Peroxidase activity, TIM44, thioredoxin reductase, pre-40s ribosomal particle, necrosis-inducing peptide 1	Detached leaves sprayed with dsRNA	[65]
	Tomato and chickpea	DCL1 and DCL2, VPS51, bik1, and SAC1	dsRNA spray	[66]
*Hyaloperonospora arabidopsis*	Arabidopsis	Hpa-CesA	dsRNA and sRNA added to spore inoculation	[67]
*Phakopsora pachyrhizi*	Soybean	Acetyl-CoA acyltransferase 40S ribosomal protein S16, glycine cleavage system H protein	Diethyl-pyrocarbonate detached leaves sprayed with dsRNA	[68]
*Plasmopara viticola*	Grapevine	PvDCL1 and pvDCL2	dsRNA sprayed post-inoculation	[69]
*Phytophthora infestans*	Potato	Sorbitol dehydrogenase, translation elongation factor 1-a, phospholipase-D like 3, glycosylphosphatidylinositol-anchored acidic serine-threonine rich HAM34-like protein, and heat shock protein-90	*E. coli* HT115 sprayed dsRNA coated with nanoclay formation	[70]
	Potato	Guanine-nucleotide-binding protein B subunit, haustorial membrane protein, cutinase, endo-1,3(4)-B-glucanase	dsRNA sprayed on detached leaves	[71]
*Sclerotinia sclerotiorum*	Barley	SsThioR, SsTlm44, SsCHC, SsAp2, SsArf72A, SsFCHO1, SsAmph, SsVATPase, and SseGFP	dsRNA clathrin-mediated endocytosis spray	[72]
	Lettuce and collard greens	DCTN1, SAC1, DCL1, and DCL2	Drop inoculation of dsRNA	[64]
	Rapeseed and Arabidopsis	Various genes involved in reactive oxygen species responses, transcription, host colonization, ribosomal biogenesis, mitochondrial protein import, and cell regulation	dsRNA sprayed on detached leaves	[65]
*Botryotinia fuckeliana*	Strawberry	Chitin synthase class Ill, DCL1, and DCL2	*E. coli*-derived minicell topical spray	[73]
*Rhizoctonia solani*	Rice	DCTN1, SAC1, and PG	Drop inoculation of dsRNA	[64]
*Aspergillus niger*	Tomato, apple, and grape	pgxB, VPS51, DCTN1, SAC1	Drop inoculation of dsRNA	[64]
*Verticillium dahliae*	Arabidopsis	DCL1, DCL2, SAC1, and DCTN1	Root dip co-inoculation of *V. dalhiae* spores and dsRNA	[64]
*Verticillium* spp.	Arabidopsis	Dicer-like DCL1 and DCL2	dsRNA co-inoculated	[62]
*Mycosphaerella fijiensis*	Banana	Nuclear condensin, coatomers alpha and zeta, DNA-directed RNA polymerase, ARP 2/3, cap methyltransferase, proteasome Pre4, ribosomal RNA, DNA polymerase alpha and delta subunits, adenylase cyclase, protein kinase C, FRQ-interacting helicase	dsRNA spore suspension	[61]
**Insect Target**	**Host**	**dsRNA Target**	**Application Method**	**Reference**
*Diabrotica virgifera virgifera*	Corn	V-ATPase A, α-tubulin, COPI coatomer	Plant dsRNA artificial diet	[74]
	Corn	Smooth septate junction (SSJ)	Artificial diet containing dsRNA	[75]
*Sitobeon avenae*	Barley	Salivary sheath protein	Naked dsRNA foliar spray of leaves	[76]
*Leptinotarsa decemlineata*	Potato	Inhibitor of apoptosis, actin, HSP70, dynamin	*Escherichia coli* HT115 dsRNA	[77]
	Potato	Actin	Naked dsRNA sprayed leaves	[78]
	Potato	Mesh gene	Naked dsRNA sprayed plants	[79]
*Phaedon cochleariae*	Cabbage	Cactus, srp54k, rop, α-SNAP shibire, PP-α, hsc70-3, rpt3	Naked dsRNA sprayed leaves	[78]
*Helicoverpa armigera*	Cotton	CYP6AE14, GST1	Plant dsRNA in artificial diet	[80]
	Chickpea	Juvenile hormone methyltransferase, acetylcholine esterase	Chitosan nanoparticles sprayed onto plants	[81]
	Cotton	CYP enzyme system	Injection of dsRNA into abdomen of fourth-instar larvae	[82]
	Cotton	HMG-CoA reductase	Injection of dsRNA into abdomen of 2-day-old female pupa	[83]
*Henosepilachna* *vigintioctopunctata*	Potato	Ecdysone receptor	*Escherichia coli* HT115 immersed in dsRNA and sprayed on foliage	[84]
*Ostrinia furnaclis*	Corn	CYP18A1, carboxylesterase	Plant dsRNA in artificial diet (dsRNA in leaves)	[85]
*Plutella xylostella*	Cabbage	Acetylcholine esterase genes AChE1 and AChE2	Plants sprayed with siRNA, taken in through insect diet	[86]
*Diaphorina citri*	Citrus	CYP4C67, CYP4DA1, CYPC68, CYPG70, CYPDB1	Insects anaesthetized and a drop of dsRNA was topically applied to the ventral side of the thorax	[87]
	Citrus	Abnormal wing disc-like protein	On 5th-instar nymphs, a drop of dsRNA was topically applied to the ventral side of the thorax	[88]
	Citrus	Arginine kinase	Foliar spray, soil/root drench, tree trunk injection, and clay soaked in dsRNA added as a soil amendment to potted citrus trees, dsRNA was ingested by insects through plant material	[89]
*Leptinotarsa decemlineata*	Potato	Actin gene	dsRNA-coated leaf surface, larvae fed for 7 days	[90]
*Halyomorpha halys*	Common bean	Juvenile hormone acid O-methyltransferase, vitellogenin	Green beans soaked in dsRNA	[89]
*Acyrthosiphon pisum*	Fava bean	Coo2	siRNA injected into insects	[91]
	Broadbean	Calreticulin	Injection	[92]
	Broadbean	Cathespin-L, vATPase	Injection and ingestion (respectively)	[93]
	Broadbean	Aquaporin	Ingestion	[94]
*Drosophila melanogaster*	Broad range	vATPase	Artificial diet containing dsRNA	[91]
*Manduca sexa*	Tobacco	vATPase	Artificial diet containing dsRNA	[93]
*Brassicogethes aeneus*	Oilseed rape	Alpha COP	Dietary exposure to buds treated with dsRNA	[95]
*Lygus lineolaris*	Cotton	Inhibitor of apoptosis gene, polygalacturonase	Injection	[96]
	Alfalfa	Polygalacturonase	Injection	[97]
*Nilaparvata lugens*	Rice	Calreticulin, cathepsin-B, NIβ2	Injection	[98]
	Rice	Trehalose phosphate synthase	Ingestion	[99]
	Rice	vATPase subunit E	Ingestion	[100]
	Rice	AGO1 and Dicer	Plant dsRNA in artificial diet (leaves soaked in dsRNA)	[85]
	Rice	NADPH–cytochrome P450 reductase (CPR)	dsRNA injection into 3rd-instar nymphs	[101]
	Rice	Calmodulins NlCaM1 andNlCaM2	dsRNA injected into nymphs	[102]
*Choristoneura fumiferana*	Spruce	Chitin deacetylase	Injection of dsRNA into larvae and pre-pupae	[103]
*Spodoptera exigua*	Beet	Chitinase7, PGCP, chitinase1, ATPase, tubulin1, arf2, tubulin2, arf1, and helicase	Injection of dsRNA into 4th-instar larvae in the abdomen	[104]
	Beet	SeCHSA	Fed dsRNA through artificial diet	[105]
	Chinese cabbage	Chitin synthase	Chinese cabbage leaf discs were soaked in guanidine coated polymer that coated dsRNA and were fed to larvae	[106]
*Spodoptera litura*	Castor	Bt toxin receptor	dsRNA injected into early 5th-instar larvae	[107]
*Spodoptera frugiperda*	Corn	sfVATPase, sfKIF, sfCDC27	Larvae fed dsRNA suspension	[108]
*Sesamia nonagrioides*	Corn	Juvenile hormone esterase-related gene	Inject dsRNA into 5th-instar larvae	[109]
*Laodelphax striatellus*	Rice	Cytochrome P450 monooxygenase Shadow (Sad)	dsRNA fed to 4th-instar larvae through artificial diet	[110]
*Aphis gossypii*	Cotton	Juvenile hormone-binding protein (JHBP) and vacuolar ATPase subunit H (V-ATPase-H)	dsRNA fed through artificial diet to first instar larvae	[111]
*Aphis glycines*	Soybean	TREH, ATPD, ATPE, and CHS1	Insect orally fed on dsRNA with a nanocarrier that was sprayed on soybean plants	[112]
*Sitobion avenae*	Winter wheat	Laccase 1	Insects fed dsRNA through artificial diet	[113]
*Tetranychus urticae*	Red kidney bean	Juvenile hormone (JH), methoprene-tolerant (Met), retinoid X receptor β, farnesoic acid O-methyltransferase, CREB-binding protein	Bean leaf discs soaked in dsRNA and fed to insects	[114]
**Plant Target**	**Host**	**dsRNA Target**	**Application Method**	**Reference**
*Nicotiana benthamiana*	Tobacco	CaMV 35S promotor	Naked dsRNA sprayed with carborundum	[115]
*Mikana micrantha*	Weeds	Chlorophyll a/b proteins	dsRNA, RNAi nanomicrosphere shRNA	[116]
*Arabidopsis thaliana*	Arabidopsis	STM and WER	Root soaking in naked dsRNA and a fluorescent nanocarrier	[117]
	Arabidopsis	EGFP and NPTII	Naked dsRNA, suspension brushed onto leaves	[118]
	Arabidopsis	Mob1A, WRKY23, actin	Root soaking in dsRNA suspension	[85]
	Arabidopsis	CHS	Leaf infiltration of dsRNA with a carrier peptide using a syringe with no needle onto plant leaf	[119]
*Dendrobium hybrida*	Dendrobium orchid	DhMYB1	Rubbing plant with bacterial extract containing the dsRNA	[120]

## 5. Spray-Induced Gene Silencing

Application of RNAi as a foliar spray has become of particular interest, specifically because it has promise in being turned into a viable and specific biopesticide that would be available for commercial use. This technology is referred to as Spray-Induced Gene Silencing (SIGS), and it is a method that allows non-transformative control of plant pests and pathogens [31,35]. This mechanism involves the application of long siRNAs and dsRNAs through a foliar spray to the affected host, which will induce RNAi when consumed by the target pest or pathogen. This mechanism can allow for specific control of harmful pathogens, without some of the downstream effects that a chemical-based pesticide may have on the surrounding ecosystem [35]. To date, SIGS has been successful in treating a wide range of pathogens and pests (Table 1). One major drawback of this technique is that ssRNAs and even dsRNAs are relatively unstable, especially when exposed to the elements [31,35]. Considerable effort surrounding the SIGS strategy is going into testing various coatings and methods to stabilize the RNAs to allow for higher efficiency [35]. 

Published exogenously applied RNA studies report efficacies of up to 100% with over 30 days of activity and were influenced by many parameters including the genetic target, RNA size, and environment (Table 1). With all of these successes, however, there are always places for improvement and many roadblocks that continue to pose challenges with SIGS. To begin with, dsRNA has relatively short environmental survival, as mentioned previously, but may be an advantage from a regulatory perspective as the molecule does not persist and contaminate the environment. Some ways of increasing this efficiency include the use of liposomes or nanoparticles as transfection agents, or even the chemical modification of one or both strands of the dsRNA [30]. Polymeric nanoparticles have been synthesized and used because of their overall stability, ease of surface modification, their biodegradability, and environmental safety. A popular polymer to use is one called chitosan, which is often used because of its relatively cheap cost, non-toxicity, and general biodegradability [30]. Taning et al. [121] reported that the use of a cationic liposome branded Lipofectamine to coat the dsRNA resulted in a 40–50% efficiency of gene-silencing. Without the use of the transfection agent, they were unsuccessful in their goal to induce gene silencing through RNAi. Chemical modifications are not often used due to their high cost and general safety concerns, but they can be used to improve molecule stability, increase double-stranded siRNA half-life in vivo, target siRNA to specific cells, and many other functions [30].

## 6. Conclusions

RNA silencing is an essential component of innate immunity and gene regulation in plants and a rapidly growing number of tools are available for applications. Efficacy and survivability of the dsRNA is being actively explored to meet specific environmental and industry requirements. Exogenous RNA survivability and uptake may be improved using polymeric, lipid-based, and inorganic nanoparticles (Table 1). Advances made in synthetic biology have opened new possibilities for optimizing traits by modulating metabolic and immunity pathways via siRNA delivery. Systemic cellular and plant movement of triggers and signals observed with the RISC response in a plant facilitates protection in tissues not exposed to the dsRNA [12,17]. As research continues on this fascinating technology, new possibilities for applications in agricultural improvements continue to emerge, such as creating improved crops that have higher yields and greater nutritional value, resistant to pests and disease, resilient to environment and climate changes, and higher yields and quality. Future developments are expected in the application of RNAi technology in plants and subsequently other biological organisms. For instance, research into controlling epigenetic elements in gene silencing will have important implications for both agricultural biotechnology and fundamental evolutionary studies [122]. There are still many exciting possibilities for future development and applications of RNA interference in the areas of crop improvement, pest management, and human health care therapies.

## Data Availability

Not applicable.
